# Pulmonary hypertension may be secondary in carriers of compound heterozygous *FOXRED1* variants

**DOI:** 10.1016/j.ymgmr.2019.100468

**Published:** 2019-03-22

**Authors:** Josef Finsterer

**Affiliations:** Krankenanstalt Rudolfstiftung, Postfach 20, 1180 Vienna, Austria

**Keywords:** Pulmonary hypertension, Respiratory chain, Complex #1, Compound heterozygous, Oxidative phosphorylation, Antioxidants

With interest we read the article by Apatean et al. about a female neonate presenting with intrauterine growth retardation, lactic acidosis, periventricular cysts, cerebral demyelination, and pulmonary hypertension(pH), being attributed to the compound heterozygous variants c.612_615dupAGTG and c.847G>A in *FOXRED1* [1]. We have the following comments and concerns.

We do not agree with the assessment of c.874G>A as “likely pathogenic” [1]. Since both parents were non-symptomatic and since the father carried only the variant c.874G>A and the mother only the variant c.612_615dupAGTG, the presence of both variants was necessary for disease expression in the index case. Thus, both variants should be assessed as pathogenic if occurring together.

Though PH is an established primary manifestation of mitochondrial disorders (MIDs) [2], PH can be also explained by the patent ductus Botalli and the patent foramen ovale. An initially left/right shunt may have been reversed after the blood volume in the pulmonary circulation had steadily increased. In addition to PH, pulmonary manifestations of MIDs include hyaline membrane disease, interstitial fibrosis, lung hemorrhaghe, restrictive pulmonary insufficiency, asthma, poor ventilator-response to hypercapnia, and obstructive sleep apnea [3]. We should know if any of these were found in the index patient.

Seizure semiology, seizure frequency, and effect of phenobarbital(PB) were not sufficiently described. We should know if the burst-suppression pattern resolved upon PB and if mitochondrion-toxicity of PB [4] contributed to the deterioration of the phenotype over time. Were antiepileptic drugs(AEDs) other than PB or the ketogenic diet ever tried?

As mentioned, dichloro-acetate(DCA) can be neurotoxic in MIDs [5]. Thus, we should be informed if the application of DCA caused any side effects and contributed to the fatal outcome.

Overall, both *FOXRED1* variants need to be assessed as pathogenic, PB and DCA may have contributed to the progression of the phenotype, and PH can be also a secondary manifestation of a MID.

## Conflicts of interest

There are no conflicts of interest.

## Funding

No funding was received.

## Author contribution

JF: design, literature search, discussion, first draft, critical comments.

## References

[1] Apatean D., Rakic B., Brunel-Guitton C., Hendson G., Bai R., Sargent M.A., Lavoie P.M., Patel M., Stockler-Ipsiroglu S. (2019). Congenital lactic acidosis, cerebral cysts and pulmonary hypertension in an infant with FOXRED1 related complex I deficiency. Mol Genet Metab Rep.

[2] Finsterer J., Kothari S. (2014). Neonatal pulmonary hypertension in mitochondrial disorders due to TMEM70 mutations. Mol Genet Metab Rep.

[3] Finsterer J., Zarrouk-Mahjoub S. (2016). Phenotypic heterogeneity of MELAS. Mol Genet Metab Rep.

[4] Finsterer J. (2017). Toxicity of Antiepileptic Drugs to Mitochondria. Handb. Exp. Pharmacol..

[5] Kaufmann P., Engelstad K., Wei Y., Jhung S., Sano M.C., Shungu D.C., Millar W.S., Hong X., Gooch C.L., Mao X., Pascual J.M., Hirano M., Stacpoole P.W., DiMauro S., De Vivo D.C. (2006). Dichloroacetate causes toxic neuropathy in MELAS: a randomized, controlled clinical trial. Neurology.

